# Changes in Patients’ Desired Control of Their Deep Brain Stimulation and Subjective Global Control Over the Course of Deep Brain Stimulation

**DOI:** 10.3389/fnhum.2021.642195

**Published:** 2021-02-24

**Authors:** Amanda R. Merner, Thomas Frazier, Paul J. Ford, Scott E. Cooper, Andre Machado, Brittany Lapin, Jerrold Vitek, Cynthia S. Kubu

**Affiliations:** ^1^Department of Psychological Sciences, Case Western Reserve University, Cleveland, OH, United States; ^2^Department of Neurology, Cleveland Clinic, Cleveland, OH, United States; ^3^Department of Psychology, John Carroll University, University Heights, OH, United States; ^4^Cleveland Clinic Lerner College of Medicine of Case Western Reserve University, Cleveland, OH, United States; ^5^Neuroethics Program, Cleveland Clinic, Cleveland, OH, United States; ^6^Department of Neurology, University of Minnesota, Minneapolis, MN, United States; ^7^Department of Quantitative Health Sciences, Lerner Research Institute, Cleveland Clinic, Cleveland, OH, United States; ^8^Center for Outcomes Research and Evaluation, Neurological Institute, Cleveland Clinic, Cleveland, OH, United States

**Keywords:** deep brain stimulation, control, ethics, neuromodulation, Parkinson’s disease

## Abstract

**Objective**: To examine changes in patients’ desired control of the deep brain stimulator (DBS) and perception of global life control throughout DBS.

**Methods**: A consecutive cohort of 52 patients with Parkinson’s disease (PD) was recruited to participate in a prospective longitudinal study over three assessment points (pre-surgery, post-surgery months 3 and 6). Semi-structured interviews assessing participants’ desire for stimulation control and perception of global control were conducted at all three points. Qualitative data were coded using content analysis. Visual analog scales were embedded in the interviews to quantify participants’ perceptions of control over time.

**Results**: Participants reported significant increases in their perception of global control over time and significant declines in their desired control of the stimulation. These changes were unrelated to improvements in motor symptoms. Improvements in global control were negatively correlated with a decline in desired stimulation control. Qualitative data indicate that participants have changed, nuanced levels of desired control over their stimulators. Increased global life control following DBS may be attributed to increased control over PD symptoms, increased ability to engage in valued activities, and increased overall self-regulation, while other domains related to global control remained unaffected by DBS.

**Conclusions**: There are few empirical data documenting patients’ desire for stimulation control throughout neuromodulation and how stimulation control is related to other aspects of control despite the growing application of neuromodulation devices to treat a variety of disorders. Our data highlight distinctions in different types of control and have implications for the development of patient-controlled neurostimulation devices.

## Introduction

Parkinson’s disease (PD) is often characterized by the acronym TRAP representing the cardinal motor symptoms: tremor, rigidity, akinesia, and postural instability. Patients with PD often use language that suggests they feel “trapped” with limited control over their body and lives due to the motor symptoms and/or unpredictable motor fluctuations. Paradoxically, in seeking to gain greater control of their symptoms and the ability to participate in valued activities and behavioral goals, patients with PD who undergo deep brain stimulation (DBS) need to relinquish some bodily control by partnering with the DBS team to share control over the brain stimulator. Patients are provided with the basic option to turn the stimulation on or off, as necessary for medical procedures, in case of emergencies, to check the batteries, or to preserve battery life in some conditions (e.g., essential tremor). Guidelines in the field advocate for maintaining constant stimulation to treat PD symptoms since motor symptoms are constant, particularly for patients implanted in the subthalamic nucleus (STN; Deuschl et al., 2006). The DBS team relies on the patient’s feedback at regularly scheduled appointments to adjust stimulator parameters similar to patients undergoing medication titration. Nonetheless, there may be differences in the patients’ perception of control of the DBS stimulator due to the invasiveness of the procedure and biotechnological properties.

This topic has generated interest in the neuroethics literature with some arguing that DBS results in self-estrangement and a loss of control vs. others who assert that DBS can enhance autonomy and control (e.g., Gisquet, 2008; Glannon, 2014; Gilbert et al., 2017). Most of the literature regarding device control *per se* has focused on patients’ perceptions of control in the context of closed-loop DBS (e.g., for a recent review see Aggarwal and Chugh, 2020) or brain-computer interfaces (BCI; see Burwell et al., 2017 for a scoping review). Data specifically addressing this question in open-loop DBS are relatively scarce. Briefly, Klein et al. (2016) conducted focus groups with eight participants and more detailed individual interviews with seven patients who were implanted with open-loop DBS systems as part of clinical trials to treat either treatment-resistant depression or obsessive-compulsive disorder. The goal of the Klein et al.’s (2016) study was to explore patients’ perspectives on closed-loop systems. One of the themes that emerged was related to control over the device function. Of most relevance to the current study, there was a range of responses regarding having control over the device with the majority of patients indicating that they would not be comfortable having sole or primary control over the stimulation and preferred that the stimulation settings be controlled by the clinical team. Goering et al. (2017) elaborated on these data and framed the participants’ responses in the context of relational autonomy. Others (e.g., Gilbert et al., 2019) relied on a phenomenological approach in a small group of patients (*n* = 6) to explore patients’ experiences with BCI in the context of treating uncontrolled seizures. Themes associated with control were evident in these data with patients indicating that the technology-enhanced feelings of control and some patients reporting the converse. Most of the available data addressing device control are qualitative data drawn from convenience samples. Reliance on convenience samples has the potential to increase bias and not reflect the experiences of the majority of patients who undergo DBS.

We prospectively examined the relationship between patients’ desired control of the stimulator settings and their perception of global life control before and following DBS surgery as part of a larger study examining patients’ goals and perceptions of control of their symptom and behavioral goals (Kubu et al., 2017). Participants were drawn from a consecutive series of patients scheduled for DBS surgery from a large academic medical center. We hypothesized that patients’ desire for control of the stimulator would increase after surgery as would their perception of global control.

## Materials and Methods

The current study was part of a larger study on patients’ goals for DBS (Kubu et al., 2017).

### Participants

A consecutive series of 59 patients approved for DBS were approached from July 2009 to June 2011 to participate in a study examining patients’ goals for DBS and perceptions of control. Most patients (*n* = 52, 88%) agreed to participate. Details regarding the patients who declined participation as well as inclusion/exclusion criteria are documented in our previous report (Kubu et al., 2017).

### Measures

All participants completed a semi-structured interview before surgery that included questions regarding their desired control of the stimulator as well as their perception of global life control. Embedded within the structured interview were visual analog scales (VAS) in which participants indicated the extent to which they desired control of their stimulator with 10 representing complete control and zero representing no control. Concerning desired stimulation control patients were asked to, “indicate (on the VAS) the degree to which they desire to control the programming (e.g., stimulation settings) of their DBS stimulation device,” and then were asked to elaborate on why they placed the mark where they did. Similarly, participants indicated the extent to which they believed they had complete control of their life (10) vs. absolutely no control (zero, similar to someone in a coma) on a separate VAS and asked to elaborate on their responses Participants completed the interview and VASs before DBS and at Post-Operative Months 3 and 6. The interview was approximately 1 h in length; it included additional questions and rating scales discussed in our previous report (Kubu et al., 2017).

Participants also completed standard clinical research outcome measures including the Parkinson’s Disease Quality of Life scale (PDQ; Jenkinson et al., 1997; Baseline, Month 6), the Unified Parkinson’s Disease Scale (UPDRS-II; Fahn and Elton, 1987; approximately Baseline, Month 3, Month 6) and UPDRS-III (Off medication at Baseline, Off medication-On stimulation 1-month post-DBS).

### Quantitative Analyses

Generalized estimating equation (GEE) models were used to examine changes over time on the VASs. Autoregressive working correlations were used for the error terms with Time as the fixed effect. Models were constructed with and without a change in the UPDRS-III as a covariate to ascertain if changes in motor symptoms significantly impacted changes in the stimulation and global life control VAS measures over time. Two sets of GEE models were constructed with the VAS variables treated as either linear or ordinal variables. The results did not change; consequently, the linear analyses are reported. Both Spearman Rho (non-parametric) and Pearson (parametric) correlations were used to assess the relationships between changes on the control measures. There was no difference in the pattern of relationships; consequently, the Pearson correlations are reported.

### Qualitative Analyses

Data from the semi-structured interviews underwent thematic content analysis to inductively and iteratively identify recurring participant-reported themes related to participants’ levels of desired control over the stimulation and their perceptions of global life control. A coding structure was developed based on recurring themes in participant interviews using content analysis by one coder. All transcripts were reviewed and large themes were identified. This was followed by a closer reading in which more nuanced and specific codes were defined that fell within those larger themes or nodes (Elo and Kyngäs, 2008). Once this coding structure was finalized, a second-rater coded a subset of the interviews to determine interrater reliability for the coding structure. Frequency distributions representing the different codes were examined at each time point to provide additional insights into participant-reported changes in desired stimulation control and global life control.

## Results

### Participant Characteristics

Fifty-two participants completed the baseline assessments. Data were available on 47 of the participants at Month 3 and 45 at Month 6 (three participants withdrew for personal reasons and the remaining four did not complete the study because they did not have surgery at our center within the study timeframe). Due to technological difficulties, interviews for six participants were not recorded; thus, those qualitative data were not available for analysis. Besides, only data from participants who completed all three research interviews were included in the qualitative analyses to allow for assessment of changes in individual participants over time. The final sample included in qualitative analyses (*N* = 39; Interview transcripts = 117) was still a sufficient sample to reach data saturation (Guest et al., 2006; Tran et al., 2017). The subthalamic nucleus was the surgical target in all but one of the participants. The participants’ demographic data, UPDRS, and PDQ scores are reported in Table 1.

**Table 1:** Demographic and Parkinson’s disease (PD) outcome measures.

Variable	Baseline *N* = 52	Post-op month 3 *N* = 47	Post-op month 6 *N* = 45
	*N* = 52	*N* = 47	*N* = 45
Gender	75% Men (*n* = 39)
Age	61.3 years (sd = 9.3)		
Duration of PD	9.1 years (sd = 4.1)		
UPDRS-II	17.2 (SE = 1.0)	12.5 (SE = 1.0)	12.0 (SE = 1.1)
UPDRS-III	38.7 (SE = 1.5)	*20.1 (SE = 1.2)	
PDQ	47.9 (SE = 3.3)		25.1 (SE = 2.5)

sd, standard deviation; SE, standard error. *UPDRS-III Off medication on stimulation scores were collected at the 1-month post-operative visit. Note: *N* = 45 for Post-op UPDRS-III; *N* = 38 for 6 month PDQ variable due to lack of post-op standard neuropsychological assessment; *N* = 41 6 month UPDRS-II variable.

### Control Ratings

**Figure 1:**
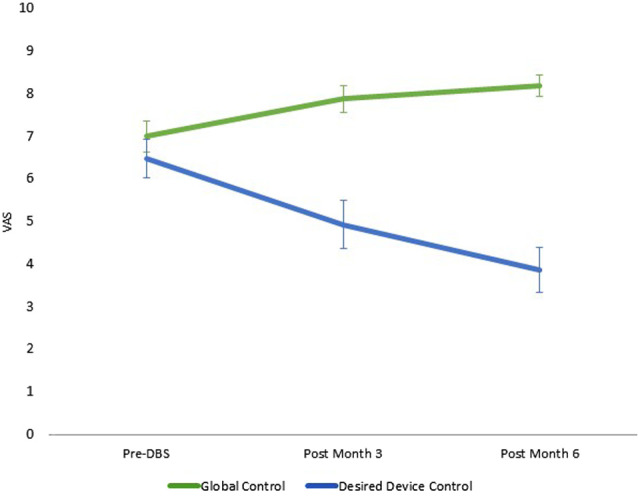
Quantitative changes in global and stimulation control over time. Note: 10, maximum control, 0, no control; vertical lines represent standard error. Data reported are the estimated means from the generalized estimating equation (GEE) models. Desired stimulator control ratings were not available at Month 3 for two participants due to examiner error.

Participants reported a significant improvement in their self-ratings of global life control ($\chi_{\left(\text{2},N=\text{144}\right)}^{\text{2}}$ = 11.11, *p* = 0.004). Similarly, significant declines in desired stimulation control were evident over time ($\chi_{\left(\text{2},N=\text{142}\right)}^{\text{2}}$ = 18.36, *p* < 0.001; Figure 1). Change in the UPDRS-III score was not a significant covariate in either model (*p*’s = 0.157, 0.879). Changes in global control were significantly correlated with changes in desired stimulator control such that as ratings of global life control increased, desired stimulation control ratings decreased over time (*r* = −0.31, *p* = 0.038). Changes in the control measures were not significantly correlated with changes in the UPDRS-III (Desired Control, *r* = −0.10, *p* = 0.518; Global Control, *r* = 0.02, *p* = 0.903).

### Qualitative Thematic Analyses

After the coding structure was finalized a second-rater coded a subset of interviews (36/117) to determine the reliability of the coding structure. Cohen’s kappa (κ) was 0.86, indicating excellent agreement (Cicchetti et al., 2006).

#### Themes Related to Stimulator Control

**Figure 2:**
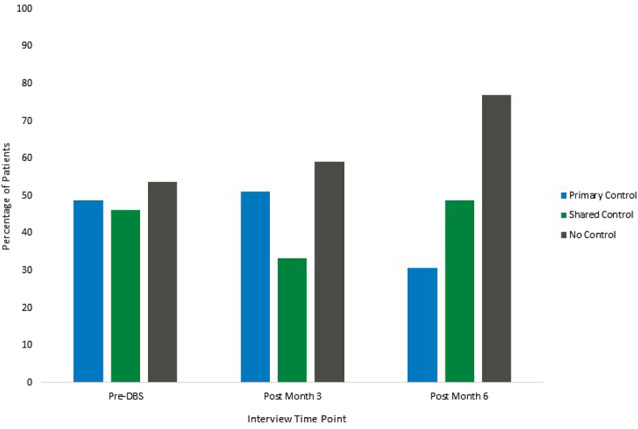
Participant themes related to desired control of stimulation. Note: participants reported more than one theme at each time point, therefore percentages of participants at each time point will add to over 100%.

Participants identified several reasons for desiring more or less control over their DBS stimulators. These themes fell under three broader categories including Primary Control, Shared Control, and No Control. Participants often identified multiple themes at each time point, therefore the percentages of participants endorsing each theme will add to over 100% at each time point. Definitions and exemplar patient quotes can be found in Table 2. Frequency distributions illustrating the relative changes in the presence of each category can be found in Figure 2.

**Table 2:** Stimulation control theme definitions and exemplar quotes.

Stimulation control themes	Definition and exemplars
Primary control	*Definition: Participant wants to be in full control of stimulator, including programming settings*. “When it comes to my body I want to control it.”>. “I would love to have full control…I’m a quick learner. I think I could learn what my body’s telling me vs. what the simulation values are fairly quickly and be able to adapt to that.”>. “I don’t want someone else having a remote telling me what to do.”>. “I’d like to be able to, if I need to dial it up or dial it down, I’d want to have the ability to do that.”>. “Well, I’d like to have control at all times. I’d like to be in charge of my life again.”
Shared control	*Definition: Participant wants to share control with DBS team, either by controlling the device themselves with assistance from the team, or providing input to the DBS team who controls the stimulator*.>. “I don’t want to control all of it because I want to have someone backing me up…I want to be able to say, “Look, I am having a problem. What can we do about this?”>. “Right now I feel a very good sense of partnership…She knows the technology, but she doesn’t know how I feel. I have to provide input.” “ “[I] just want to be able to communicate with them how well it’s doing, if it needs to be adjusted up or down or whatever would be my input.” “I expect this would be a 50/50 adventure. If I have a problem with where it’s set I want to be able to tell them that and get some serious consideration about changing it.”
No control	*Definition: Participant wants to have no control over the stimulator (beyond basic ON/OFF) and the DBS team controls all aspects of the device programming*.>. “I don’t want any [control]. I want the doctor to do it.”>. “I don’t really want control of it. I’d rather leave that up to the professionals who know what they’re doing.”>. “I don’t have any desire to control the settings on it at all. I don’t think I’m qualified to do any of that at all. I think I have a lot of faith and confidence in the technicians to do that. That’s their job, not mine.”>. “I want no control because she [programmer] does it and that’s working great.”

##### Primary Control

Several participants discussed reasons for desiring primary control over their stimulators. At the baseline interview, which took place before surgery, 48.7% of the participants discussed themes indicating their desire to have primary control of the stimulator. Themes in this category included participants desiring control over their bodies, wanting the ability to adjust parameters to control fluctuating symptoms, eliminating or reducing the amount of travel and number of visits to receive programming, and several participants felt confident they could be trained to program their stimulators if given the proper education. The percentage of participants endorsing themes related to primary control of the stimulator remained relatively stable from the baseline interview to the first post-surgical interview at 3 months (51.2%) and decreased at the final 6-month interview (30.8%).

##### Shared Control

Participants also identified several themes that demonstrated a desire to have a partnership with the surgical team and programmer, with some individuals desiring to control stimulator settings with the team’s guidance, and others desiring their input to be used to guide programming. Several individuals also expressed satisfaction with having the ability to turn the stimulator on and off and check batteries, while leaving the programming in the hands of the team. At the baseline appointment, 46.2% of the participants discussed the desire for some form of shared control between the participant and the DBS team. The percentage of patients who discussed themes related to shared control decreased from baseline to the 3-month interview (33.3%) and increased at the 6-month interview (48.7%).

##### No Control

Participants discussed several reasons for desiring no control over the stimulation. These themes were related to trust in the team and the team’s expertise, as well as satisfaction with how the stimulator was working. Participants also discussed their apprehensions about having control over their stimulators, with many saying they would not want to harm themselves or break the stimulator, and stating they were not qualified to program the stimulator and they do not want that responsibility. At the baseline appointment, 53.8% of the participants discussed themes related to having no desire to control the stimulation. The percentage of participants that discussed these themes increased slightly at the 3-month interview (59.0%) and increased further at the 6-month interview (76.9%).

#### Themes Related to Global Life Control

**Figure 3:**
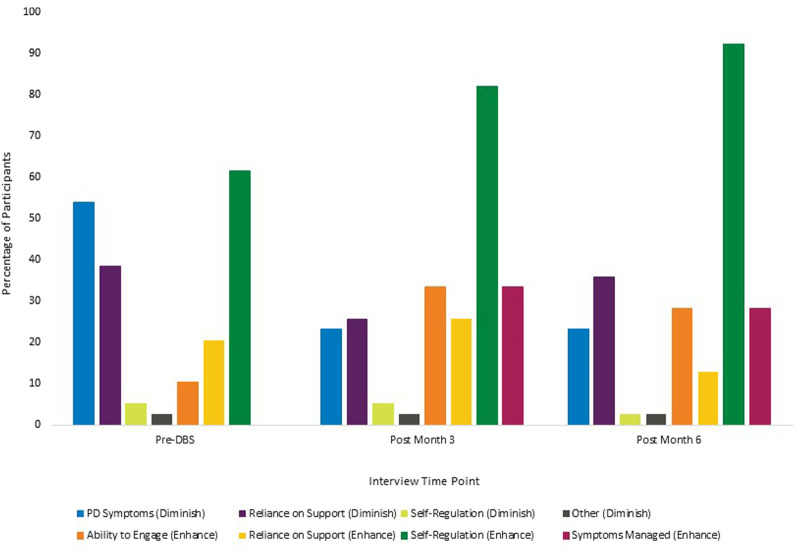
Participant themes related to global life control. Note: participants reported more than one theme at each time point, therefore percentages of participants at each time point will add to over 100%. Categories that contained themes related to both enhanced control and diminished control have been divided and reported separately in the figure.

Participants identified many different aspects of their lives that contributed to enhancing or diminishing their feelings of global life control. These themes fell under six broader categories including Parkinson’s Disease Symptoms and Challenges (diminish control), Reliance on Support Systems (mixed effects on control), Internal Self-Regulation (mixed effects on control), Continued Ability to Engage in Activities (enhance control), Symptoms Managed/General Health (enhance control), and Other (diminish control). Frequency distributions illustrating the relative changes in the presence of each category can be found in Figure 3.

##### PD Symptoms and Challenges

Participants identified several ways in which PD symptoms and challenges diminished their overall level of global life control. Themes in this category included fluctuating PD symptoms, ways in which the various PD symptoms make patients’ lives more challenging, participants feeling as though PD has taken over their bodies, and an awareness that PD is a progressive disease without a cure so their condition will continue to worsen. At the baseline interviews, 53.8% of participants discussed themes in this category. After DBS surgery, the presence of these themes in participant interviews decreased, with 23.1% of participants discussing themes related to PD symptoms and challenges at 3 months and 23.1% again at 6 months.

##### Reliance on Support Systems

Participants identified several ways in which reliance on various support systems either enhanced or diminished their feelings of global life control. These themes have been separated into enhancing or diminishing control in Figure 3 for ease of interpretation. Participants discussed themes surrounding the notion that God is in control of their lives and how their reliance on others to help with daily activities, the DBS stimulator or medication, or reliance on the programmer or DBS team diminish the sense of control. At baseline, 38.5% of participants discussed themes related to ways in which reliance on support systems diminished their feelings of global control. The percentage of participants endorsing these themes fluctuated after surgery, with 25.6% of participants discussing these themes at the 3-month interview and 35.9% at the 6-month interview. In contrast, participants identified several themes in this category that enhanced participants’ perceptions of control included having a good support system of friends and family, feeling more in control because God is helping them, and the impact of the stimulator or medications in restoring control. At baseline, 20.5% of participants discussed themes related to how reliance on support systems enhanced feelings of global life control. After surgery, the percentage of patients discussing these themes fluctuated, with an initial increase at the 3-month interview (25.6%) and then a decrease at the 6-month interview (12.8%).

##### Internal Self-Regulation

Participants identified several ways in which aspects of internal self-regulation either enhanced or diminished their feelings of global life control. These themes have been separated into enhancing or diminishing control in Figure 3 for ease of interpretation. Themes in this category that diminished perceptions of control included participants’ feelings of uncertainty regarding their physical limitations and feelings of anxiety or fear when trying to engage in different activities. At baseline, 5.1% of participants discussed themes related to ways in which internal self-regulation diminished their feelings of global control. The percentage of participants endorsing these themes remained stable after surgery, with 5.4% at the 3-month interview and then a decrease to 2.6% at the 6-month interview. Themes related to internal self-regulation that enhanced participants’ perceptions of control included being cognitively in control, having the ability to make important decisions in their lives including the decision to seek different treatment options, being in control of their outlook and attitude, feeling an overall sense of independence, having control over when they ask for help and being able to communicate how they feel, and having less fear and anxiety about physical limitations. At baseline, 61.5% of participants discussed themes related to how internal self-regulation enhanced feelings of global life control. After surgery, the percentage of participants discussing these themes increased at the 3-month interview (82.1%) and increased again at the 6-month interview (92.3%).

##### Continued Ability to Engage in Activities

Participants discussed their ability to engage in various activities as something that enhanced their feelings of global life control. Activities included engaging in personally-meaningful hobbies, working and volunteering, and interacting with friends and family members. At the baseline interview, 10.3% of the participants discussed themes that fell into this category. After surgery, the percentage of participants endorsing these themes fluctuated, with an initial increase to 33.4% at the 3-month interview and then a decrease to 28.2% at the 6-month interview. It is worth noting that although two fewer participants discussed themes related to their continued ability to engage at the 6-month interview, this remains an overall increase from pre-DBS to post-DBS.

##### Symptoms Managed/General Health

Participants cited feeling generally healthier, having better control of their symptoms, and feeling more in control of their bodies as reasons for enhanced feelings of global life control. At baseline, none of the participants discussed these themes. However, after surgery, 33.4% of the participants discussed themes in this category in the 3-month interview and 28.2% of the participants identified these themes at the 6-month interview.

##### Other

There were only three total instances (less than 1% of themes present at all of the time points) when participants provided reasons they felt their global life control had been diminished that did not fit into the existing coding structure. These included the need to continue to work and responsibilities for others, both of which resulted in perceived decreased control.

## Discussion

Participants reported decreases in their desired control of stimulation throughout DBS treatment. Simultaneously, participants reported significantly greater global control over their lives. The changes in desired stimulation control and global life control were negatively correlated such that as desired stimulation control declined, the participants’ perception of global control increased over time. Quantitative findings demonstrate that changes in control ratings were unrelated to improvements in the patient’s motor symptoms as measured using the UPDRS-III. This is the first report, to our knowledge, that systematically assessed a large, consecutive series of PD patients’ desire for stimulator control as well as the perception of global control throughout DBS treatment.

The qualitative responses from the patients provide insight into factors that influenced the changes in the control ratings. Many patients indicated that their reduced desire to control the DBS stimulator, including stimulator settings, reflected a sense of collaboration with, trust in, and respect for the DBS team’s expertise. These findings are very similar to those documented in the work by Klein et al. (2016) and support a relational autonomy framework as articulated by Goering et al. (2017). For example, many patients indicated that their input regarding stimulation effects was critical in helping the team optimize stimulation. Some patients also indicated that they felt that turning the stimulator on and off was sufficient control for them and they relied on the team for controlling other aspects of the stimulation.

A review of the qualitative global control data indicated that increases in global life control may be partially attributed to a reduction of PD symptoms, a finding that contradicts our findings that improvements in global control were unrelated to changes on the UPDRS-III. This discrepancy highlights the importance of approaching these questions using a mixed-methodology to gather a more holistic view of the participants’ experiences and also illustrates that the standard clinical outcome measures used to assess treatment efficacy, such as the UPDRS-III, may not fully capture patients’ experiences (see Kubu et al., 2017). The reduction in PD symptoms also came with an increase in the number of participants citing their ability to engage in valued activities as a reason for increased perceptions of global life control. Although these factors are important drivers of change in ratings of global life control from pre- to post-DBS surgery, participants also highlighted other themes that impacted their sense of control. Our data demonstrate that beyond the management of PD symptoms, participants rely on their relationships with others (including God, family, and the DBS team) once again highlighting the relational aspect of control, as well as their ability to internally self-regulate across cognitive, affective, and interpersonal domains to maintain a sense of control over their lives. Participants reported their reliance on relationship supports remained relatively unchanged before and after DBS surgery, meaning the surgery does not diminish their control in this highly personally relevant domain. Further, the qualitative data demonstrate an overall increase in the percentage of patients who discuss enhanced internal self-regulation, with 36 of the 39 participants (92.3%) endorsing themes related to feeling a sense of independence at the 6-month interview compared to 61.5% before surgery. Taken together these findings indicate that participant-identified themes related to relationships and the belief in one’s own ability to control one’s behavior work in conjunction with improved symptom management for an overall increased sense of global life control.

These findings are limited by the relatively brief follow-up period. It may be that patients’ desire to control the stimulator may decline even more over time as they habituate to the stimulator or patients’ desire to control the stimulator may increase as symptoms progress. It is also possible that feelings of control may change as the need to undergo battery replacements arise. Second, participation in a study specifically designed to explore patients’ expectations surrounding control may have resulted in a positive bias toward the team resulting in greater trust and/or willingness to share control with the team. Third, these data represent patients’ desired stimulation control when actual stimulator control was limited to turning it on/off. In our center, rarely, DBS patients with PD would regularly choose or be advised to turn their stimulator off. This is consistent with expert guidelines in the literature (Deuschl et al., 2006) and reflects the fact that most DBS candidates with PD can experience their primary motor symptoms constantly if not treated. Thus, although they had control to turn the stimulator on or off, most participants would be unlikely to exercise that option. Nonetheless, even in this simple example, our data highlight the need to study patients’ preferences for stimulation control throughout DBS as those preferences may change, and what patients define as primary, shared, and no control can change as they learn more about the stimulator and experience DBS. For example, several patients identified having the ability to turn their stimulators on/off as having no control at the baseline interview, but by the end of the study felt this ability gave them primary control over the stimulator. Future studies should follow patients for longer periods, include other centers, and compare devices that offer differing options for patient control of stimulation to further explore and understand how desired control of the stimulator settings and perceptions of global control over one’s life are related and, if our findings are replicated, what drives those relationships. Finally, although our participants reflect the gender demographics of PD and are similar to other largescale outcome DBS studies, our sample was heavily skewed toward Caucasian men. Consequently, these findings should not necessarily be generalized onto other demographic groups whose sense of control may be influenced by sociocultural factors related to gender, ethnicity, and race^1^. Similarly, these findings should not be generalized to other patient groups with different disorders and stimulation targets since all of these important variables may influence patients’ perceptions of control (Kubu et al., 2019).

Despite these limitations, our findings suggest that reductions in desired stimulation control do not correspond to parallel reductions in perceived global control over one’s life in the context of DBS for the treatment of motor symptoms in PD. These data highlight the important distinction between different aspects of control and suggest that patients may be more willing to share or cede one aspect of bodily control (i.e., changing stimulation settings of an implanted brain device) to the medical team as they gain greater global control over their lives following DBS surgery. We hope that these empirical data can help inform future conceptual, neuroethical analyses which are beyond the scope of this brief report. Our data provide support to the perspectives that DBS can supplement a patient’s sense of autonomy and control *via* a model of shared control (Glannon, 2014) or relational autonomy (Goering et al., 2017). The data are also consistent with Klein et al.’s (2016) observations that most patients in their sample preferred to defer control of the stimulation parameters to the medical experts. Also, our data illustrate the importance of recruiting a consecutive series of patients to obtain a better understanding of most patients’ experiences. Finally, our findings also have implications for the development of patient-controlled neuromodulation devices and highlight the importance of assessing patients’ perceptions surrounding control throughout DBS. Quite simply, patient-rated measures collected before surgery may not reflect patients’ rated stimulation control preferences after they have experienced DBS.

## Data Availability Statement

The raw data supporting the conclusions of this article will be made available by the authors, without undue reservation.

## Ethics Statement

The studies involving human participants were reviewed and approved by Institutional Review Board, Cleveland Clinic. The patients/participants provided their written informed consent to participate in this study.

## Author Contributions

ARM, TF, and BL: data analysis and interpretation, writing and critical revision of the manuscript. SC, AM, JV, and PF: study concept and design, writing and critical revision of the manuscript. CK: study concept and design, data analysis and interpretation, writing and critical revision of the manuscript. All authors contributed to the article and approved the submitted version.

## Conflict of Interest

The authors declare that the research was conducted in the absence of any commercial or financial relationships that could be construed as a potential conflict of interest.

## Footnotes

^1^We are grateful to an anonymous reviewer for raising this important point.
